# Response of grassland ecosystem to monsoonal precipitation variability during the Mid-Late Holocene: Inferences based on molecular isotopic records from Banni grassland, western India

**DOI:** 10.1371/journal.pone.0212743

**Published:** 2019-04-17

**Authors:** Sayak Basu, Prasanta Sanyal, Anusree A. S. Pillai, Anoop Ambili

**Affiliations:** 1 Indian Institute of Science Education and Research Kolkata, Mohanpur, West Bengal, India; 2 National Centre for Biological Sciences (NCBS), GKVK Campus, Bangalore, Karnataka, India; 3 Manipal Institute of Higher Education, Madhav Nahar, Manipal, Karnataka, India; Durham University, UNITED KINGDOM

## Abstract

Banni, located in the arid western India, is one of the largest tropical grasslands of the Asian continent. The net primary production in this grassland ecosystem is currently mediated by precipitation during the Indian summer monsoon (ISM). However, timing of the grassland expansion and its link to the intensity of monsoonal precipitation remains enigmatic due to the paucity of datasets. The major objective of this study is to understand the changes in monsoonal precipitation and vegetation for the last 4600 cal yr BP using hydrogen and carbon isotopic composition of *n*-alkanes (δD_*n*-alkane_ and δ^13^C_*n*-alkane_) measured from two core sediments (Chachi and Luna) in Banni region. The δ^13^C_C29_ and δ^13^C_C31_ values for Chachi core sediments vary from −30.9 ‰ to −27.2 ‰ and −34.4 ‰ to −25 ‰ respectively. The δ^13^C_*n*-alkane_ values from the core sediments are converted into %C_4_ plants based on a binary mixing model using the end-member δ^13^C_*n*-alkane_ values derived from the dominant modern vegetation in the Banni region. The prominent feature of the paleovegetation curve is the marked increase in the δ^13^C_*n*-alkane_ values after 2500 cal yr BP, which suggests proliferation of C_4_ grasses in this region. Similar changes after 2500 cal yr BP have also been observed in the δD_*n*-alkane_ values. The δD_C29_ values are used to calculate δD value of paleoprecipitation that varied from 10 ‰ to −60.2 ‰. A significant increase in the δD values of paleoprecipitation (ca. 25 ‰) indicates a weakened ISM precipitation after ca. 2500 cal yr BP. The regional aridification and frequent fire events may have helped the expansion of C_4_ plant dominated grassland ecosystem in Banni region. Correlation between paleoclimatic records suggests that the southward migration of intertropical convergence zone and more frequent warm phases of El-Nino Southern Oscillation have triggered the weakening of monsoonal precipitation in the tropical region.

## Introduction

Grasslands are globally important as they comprise nearly one-fifth of the world’s land surface and 80% of the total agriculturally productive land [1–2]. Geographical distribution, community composition and net primary production of grassland ecosystems not only play crucial roles in global carbon budget [3] but also influence regional climate by modulating the evapotranspiration flux [4]. The vegetation composition of grassland ecosystems reflects the competing influence of precipitation, fire and atmospheric CO_2_ concentration. For instance, changes in CO_2_ level and fire events in the grassland of West Africa during the Late Holocene shifted the vegetation composition towards woody plant cover, despite the increased regional aridity [5]. It is important to note that the interaction between above-mentioned forcing factors varies across the globe and thus produces a site-specific structure of the grassland ecosystem. Furthermore, the magnitude of the climatic factors and their effects on vegetation composition are also globally variable, depending upon duration of the rainy season, precipitation amount and number of precipitation events [6]. Prolonged and recurrent droughts predicted in a warming environment, combined with human-induced changes in the natural hydrological regime, will likely have an adverse effect on biodiversity of the grasslands [7, 8]. The understanding of past climate-ecosystem coupling is thus vital to predict future ecosystem response to changing natural variables.

The available Mid-Late Holocene paleoclimate records from Indian summer monsoon (ISM) domain suggest significant modifications in seasonal distribution of monsoonal precipitation in response to shifts of the Intertropical Convergence zone (ITCZ) [9–11]. Changes in hydrological regime will likely to have a major impact on the biodiversity of the Banni grasslands in western India—one of the largest tropical grassland of Asian continent. The modern day precipitation of Banni grassland is characterized by extremely sparse rain events (n = 10–12), short length of monsoon season and very high temperature difference (ca. 40 °C) between summer and winter months. This extreme climate setting makes the Banni grassland an ideal site to investigate the past climate-grassland interactions.

A previous study from the Banni grassland used the carbon isotopic composition of bulk organic matter (δ^13^C_org_) and oxygen isotopic composition of gastropod shells (δ^18^O_shell_) to decipher paleoenvironmental changes [12]. The δ^13^C_org_ values from the Banni grassland suggested Late Holocene expansion of a C_4_ grass dominated ecosystem in response to changes in moisture availability and local fire-events [12, 13]. However, δ^13^C_org_ value based estimates of %C_4_ plants have uncertainties due to thermal degradation of selective organic compounds [14], which can alter the pristine isotopic character [15]. Furthermore, δ^13^C_org_ values integrate organic matter from numerous sources (each with distinct δ^13^C value) that can often led to bias in paleovegetation reconstruction [16]. Likewise, uncertainty has also been associated with δ^18^O_shell_ based climate interpretation due to interplay of factors such as local precipitation variability, changes in the moisture pathway, lake water evaporation, residence time, temperature as well as biological effects controlling the δ^18^O values [17]. Therefore, more robust hydrological and vegetational proxies are required for an improved assessment of the paleoenvironmental changes from this region.

**Fig 1:**
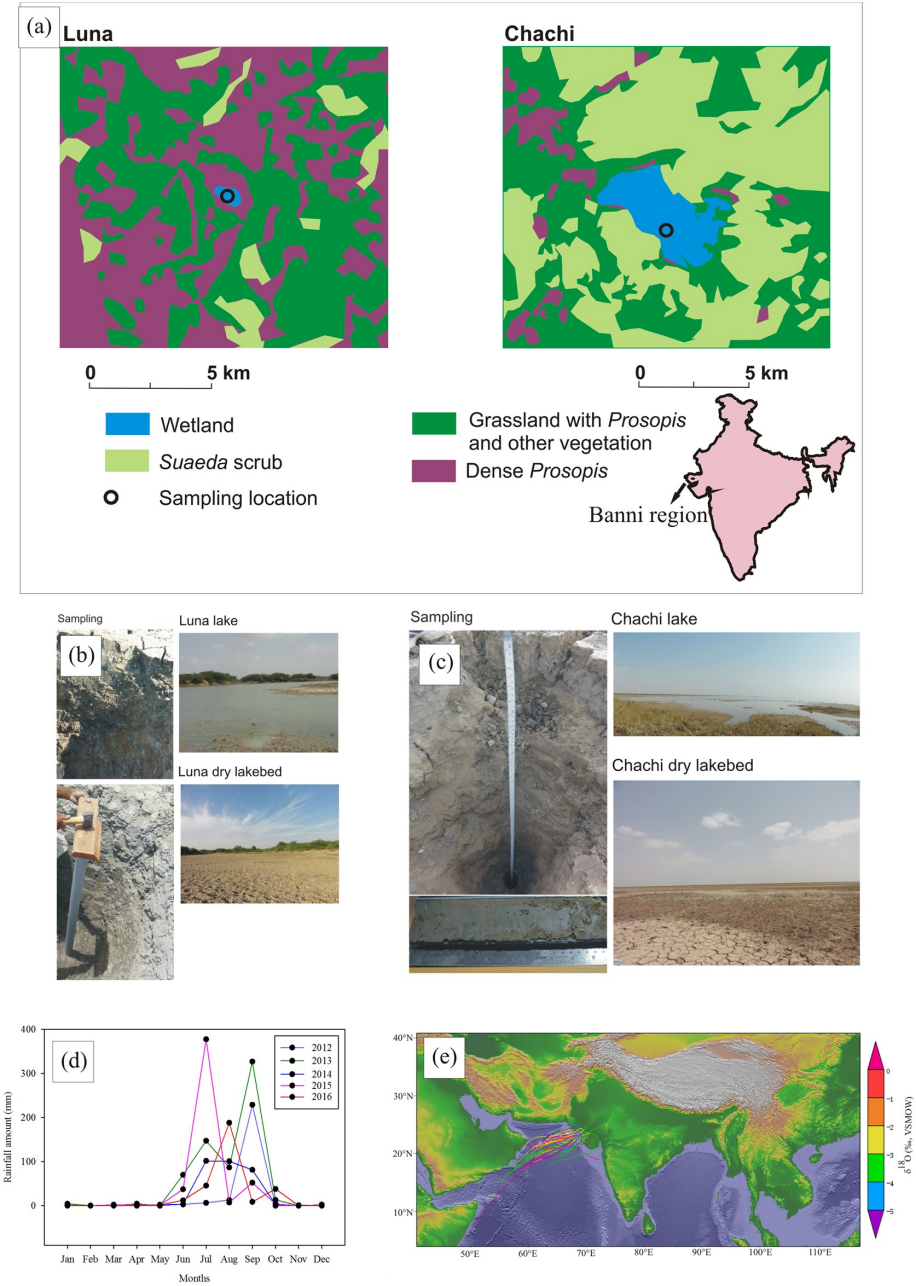
Study area, sampling and modern climate in Banni grassland. (A) Schematic representation of plant functional types around the seasonal wetlands [13, 18]. Images showing core sampling in (B) Chachi and (C) Luna wetlands. (D) Monthly precipitation in the Banni region for past 5 years. (E) Back-trajectory analysis of the air-parcel demonstrates that Arabian Sea is the dominant moisture contributor to the precipitation in Banni region.

To achieve a better understanding of the past vegetation composition and its linkage with climatic factors, the present study measured carbon and hydrogen isotopic composition of *n*-alkanes (δD_*n*-alkane_ and δ^13^C_*n*-alkane_) extracted from two seasonal wetland sediment cores (Chachi and Luna) (Fig 1). The relative phasing of vegetation and precipitation are assessed from the isotopic composition of same compound (i.e., *n*-alkane). Comparison of records from multiple seasonal wetlands provides a synoptic view of past environmental changes in this region. The objectives of the present study include (i) understanding the potential sources of organic matter (OM) in the Banni region; (ii) reconstruction of the climatic conditions and local vegetation history; (iii) recognizing the spatial variability of ISM; (iv) interpreting the role of teleconnections on the intensity of ISM precipitation and; (v) understanding the feedback mechanism loop between monsoonal precipitation, fire and CO_2_. To our best of knowledge, this is the first continuous biomarker-based record of climate change from western India during the Mid-Late Holocene.

## Study area

### Modern climate

The Banni terrain (total area: 3,847 km^2^, 23°19' to 23°52' N and 68°56' to 70°32' E) is a flat transitional land which lies between the mainland Kachchh and the Great Rann, one of the world’s largest hypersaline marshy area. This region accounts for 8.4% of the total area of Kachchh Desert, western India [19]. The Banni region receives the dominant fraction (80–90%) of annual precipitation (avg. 310 mm) during ISM (Fig 1D). The average seasonal temperature in this region varies from 50 °C in summer (May-July) to 10 °C in winter (December-February) [18].

### Geomorphology

Banni, a tectonically raised mudflat, is located on the southern edge of the Great Rann of Kachchh [20]. This unique geomorphic terrain is slightly elevated (ca. 4 to 20 m) from the Great Rann with sediments comprising of fluvial origin [20]. The sediments in the Banni region are derived from the Mesozoic sedimentary rocks of northern Kachchh Mainland brought down by the north flowing drainage system comprising of Khari, Chhari and Kaila rivers [21–22]. The fine textured Banni sediments are composed of stratified silt and clay with thickness more than 30 m [23]. Soil salinity is highly variable, and pH ranges between 6.5 and 8.5 [23].

### Modern vegetation

The modern-day vegetation in Banni region is characterized by the mixed C_3_-C_4_ plants [13] (Fig 1A). Vegetation of Banni comprises of herbs (89 species, accounts for ca. 46% of all the plant species), grasses (37 species, ca. 19% of all the plant species,) shrubs (31 species, ca. 16% of all the plant species), trees (17 species, ca. 9% of all the plant species), and sedges and climbers (ca. 10% of plant species) [24]. The Chachi site comprises of Suaeda scrub and grassy vegetation with sparse cover of *Prosopis juliflora* and other herbaceous vegetation, whereas the catchment of Luna is composed of grassland with moderate density of *Prosopis juliflora* [13]. The region is covered with coarse and low perennial grasses like the *Desmostachya bipinnata*, *Sporobolus marginatus*, *Dichanthium annulatum*, *Cenchrus ciliaris*, *Sporobolus fertilis* and *Chloris barbata* [25]. Prominent tree species of this region are *Acacia nilotica subsp*. *indica*, *Prosopis juliflora*, *Prosopis cineraria*, *Salvadora oleoides and Salvadora persica* [25]. The region is currently degraded mostly due to frequent droughts events coupled with intense grazing and invasion of *Prosopis juliflora* [19].

## Methods

### Field sampling

Core sediments were collected from two closed shallow seasonal wetlands (Chachi and Luna) during a field expedition in summer 2012 when the region was completely dried up (Fig 1B and 1C). The cores (ca. 138 cm and 85 cm depth for Chachi and Luna, respectively) were retrieved using 6 cm diameter, long PVC pipes. No specific permissions were required for the sample collections from these locations. All the samples were stored at 4 °C before the analysis. The sediment cores collected from Chachi and Luna were clayey with occasional silt laminations [12]. The Chachi core was sub-sampled at 5–8 cm intervals until 70 cm depth followed by 4 cm intervals and Luna core was sub-sampled at 6 cm intervals. In addition to core sediment samples, leaf samples of dominant vegetation (grasses, herbs and trees) were also collected. The surface of the leaf samples were cleaned with 0.5 N HCl and distilled water in an ultrasonic bath to remove adhered contaminants. Leaf samples were dried at 40 °C for 48 hours, and core sediment samples were dried at 50 °C for 8 hours. Leaves and sediment samples were subsequently powdered in an agate mortar for further analysis. All the biomarker-based analyses were conducted at the stable isotope laboratory of Indian Institute of Science Education and Research Kolkata.

### Extraction and analysis of *n*-alkane

Total lipid extract (TLE) is obtained via accelerated solvent extractor (Dionex, ASE350) using dichloromethane/methanol (93:7) mixture at 100 °C and 1600 psi pressure for 15 min (2 cycles). For this purpose, 0.3–0.5 g leaf and 7–8 g sediment samples were used. Sample preparation and lipid extraction methods have been described in detail elsewhere [12, 26, 27]. TLE fraction was concentrated in a Rotavapour (R-210, Buchi) by evaporating the dichloromethane and methanol mixture. The non-polar hydrocarbon fraction (*n*-alkane) was separated from the TLE using short-column silica gel column chromatography, using activated silica gel (100–200 mesh) in Pasteur pipette plugged with glass wool and hexane as eluent. Then *n*-alkane fraction has been concentrated up to 0.5 ml under a stream of dry N_2_.

The concentration of individual *n*-alkane was measured in a gas chromatograph (Agilent 7890A, GC system) equipped with a split/splitless injector, non-polar capillary column (HP5-MS, 30 m × 250 μm × 0.25 μm) and flame ionized detector (FID). Samples were injected in 1:1 split mode with an initial inlet temperature of 320 °C. The GC oven temperature was started at 60 °C (held 2 min) and then increased to 320 °C at 8 °C/min (held 12 min). The characteristic retention time obtained from the external *n*-alkane standard mixture (SUPELCO C_8_–C_40_ alkanes) was used for the identification and quantification of individual *n*-alkanes.

### The *δ*D and *δ*^13^C analyses of *n*-alkane

For the Chachi core sediments, a total of 19 and 16 samples were measured for δ^13^C_*n*-alkane_ and δD_*n*-alkane_ values respectively. For Luna core sediments, δ^13^C_*n*-alkane_ analysis has been carried out on 14 samples. All samples were not analyzed for δD_*n*-alkane_ values compared to δ^13^C_*n*-alkane_ due to limited sample material.

The δ^13^C_*n*-alkane_ values were measured in Thermo Trace GC Ultra, connected via combustion interface (GC-Isolink) and Conflo IV interface to a MAT 253 isotope ratio mass spectrometer (GC-IRMS). The GC oven temperature was raised from 40 °C (held 1 min) to 320 °C (held 12 min) at 10 °C/min. 1 μl samples were injected in splitless mode with an inlet temperature at 280 °C and He as carrier gas at 1 ml/min. Individual compounds were combusted over Nickel and Copper wire in the presence of O_2_ with He (1% v/v) at 950 °C to produce CO_2_. Calibration of δ^13^C_*n*-alkane_ value was performed by injecting several pulses of CO_2_ at the beginning and the end of each GC run. Isotopic ratios are expressed in ‰ relative to the Vienna Pee Dee Belemnite (VPDB) standard. A standard mixture of *n*-alkanes (C_16_-C_30_) procured from Prof. Arndt Schimmelmann of Indiana University, with a known isotopic composition was used at different dilution (0.5, 1, 1.5, 7.5 and 15 ng/μl) to monitor the accuracy of the measurement.

The δD_*n*-alkane_ values were measured using a Thermo Trace GC Ultra coupled via a GC Isolink pyrolysis interface operated at 1420 °C to a Thermo MAT 253 GC-IRMS. The δD_*n*-alkane_ values were measured against calibrated H_2_ reference gas. The δD_*n*-alkane_ values are reported in ‰ notation against the Vienna Standard Mean Ocean Water (VSMOW). The Schimmelmann standard was used at different dilution (35, 50, 75 and 105 ng/μl) to assess the accuracy of the analysis. Long-term repeated analysis of the external standard mixture rendered a precision of ±3 ‰ (1σ) and an average accuracy of 1 ‰. The H_3_^+^ factor varied around 11.1 ± 0.02 ppm nA^-1^ throughout the measurement period.

### Chronology

The published ^14^C activity measurements made on bulk organic matter are used to constrain an age model for Chachi and Luna core sediments [12]. However, using the radiocarbon activity of bulk organic carbon to date the deposition of lake sediments is often hampered by i) the “reservoir effect”, carbon in the organic fraction derived from old groundwater or dissolution of limestone [28, 29, 30]; (ii) contamination by reworked organic materials from weathering and erosion in the upstream catchment can dilute the ^14^C content of organic materials with respect to the atmosphere at the time of deposition [31, 32]. The chronology based on the AMS dating of bulk organic sediments in Chachi and Luna core sediments are stratigraphically consistent [12]. The bulk organic matter in the Banni core sediments has dominant source derived from the terrestrial vascular plants [12] that incorporate ^14^C in cellulose sourced from ^14^CO_2_ in the atmosphere [33]. Therefore, the bulk organic matter in the Banni core sediments is relatively free of any “reservoir effect” making it an ideal material for applying ^14^C to date the sediments. The ^14^C dates were calibrated using OxCal 4.1 software [34] with the IntCal 09 calibration curve [35]. The average sedimentation rates of the Chachi and Luna sediments are 0.03 and 0.06 cm yr^-1^ respectively. The age-depth relationship was modeled using linear interpolation between adjacent radiocarbon measurements.

### Quantification of organic source using *n*-alkane indices

The *n*-alkane indices have been used to obtain information on OM sources using the source-specific carbon chain length [36, 37]. The long-chain *n*-alkanes (C_27_–C_33_) tend to dominate terrestrial leaf waxes, short-chain *n*-alkanes less than C_21_ are more characteristic of algae, and mid-chain *n*-alkanes (C_21_, C_23_, and C_25_) are mainly produced by aquatic macrophytes [38, 39].

In the present study, P_aq_, Terrigenous versus aquatic ratio (TAR) and carbon preference index (CPI) have been calculated. The P_aq_ and TAR indices have widely been used to estimate the relative proportion of terrestrial flux in the sediments [39, 40].

Following equations have been used in this study:

$$\text{Paq}=\frac{\left({\text{C}}_{23}+{\text{C}}_{25}\right)}{\left({\text{C}}_{23}+{\text{C}}_{25}+{\text{C}}_{29}+{\text{C}}_{31}\right)}$$

$$\text{TAR}=\frac{\left({\text{C}}_{27}+{\text{C}}_{29}+{\text{C}}_{31}\right)}{\left({\text{C}}_{15}+{\text{C}}_{17}+{\text{C}}_{19}\right)}$$

$$\text{CPI}=0.5\times \left[\frac{\left({\text{C}}_{27}+{\text{C}}_{29}+{\text{C}}_{31}+{\text{C}}_{33}\right)}{\left({\text{C}}_{26}+{\text{C}}_{28}+{\text{C}}_{30}+{\text{C}}_{32}\right)}+\frac{\left({\text{C}}_{27}+{\text{C}}_{29}+{\text{C}}_{31}+{\text{C}}_{33}\right)}{\left({\text{C}}_{28}+{\text{C}}_{30}+{\text{C}}_{32}+{\text{C}}_{34}\right)}\right]$$

[41].

### Back-trajectory computation

The probable moisture source of the air masses has been deduced based on the backward trajectories computed from the database of the National Oceanic and Atmospheric Administration (NOAA, http://www.arl.noaa.gov/ready/hysplit4.html) using Hybrid Single-Particle Lagrangian Trajectories (HYSPLIT-4). Trajectories were computed for 48 hours at 500, 1500 and 2500 m.a.s.l. as precipitation in India is expected to originate from these altitudes [42]. In daily time-scale, trajectories did not show much variation with altitude, and hence 500 m.a.s.l. was considered to be cloud base height for precipitation over the Banni region.

### The δD value of precipitation in Banni grassland

In the absence of meteorological station in Banni grassland, the Isotope Global Spectral Model version 2 (IsoGSM2) prediction of the stable isotopic composition of regional precipitation [43] has been used in the present study. This model provides monthly averaged δD and δ^18^O values of precipitation for the years 2011 to 2015. The spatial resolution of the model is 1.9° latitude 1.8° longitude. In IsoGSM2, atmospheric processes and temperature fields are constrained by spectral nudging. The detailed description of the model has been provided elsewhere [43]. In this study, 5 years weighted averaged δD and δ^18^O values of precipitation have been used as a reference dataset to interpret paleoclimatic signals.

### Statistical analyses

The Mann-Whitney U test has been used to assess statistically significant differences between δD_*n*-alkane_ and δ^13^C_*n*-alkane_ values of the Chachi core sediments before and after 2500 cal yr BP. This test is commonly used to identify the significantly differing groups, which are not normally distributed [44]. The null hypothesis of this non-parametric test is that the difference of location between the samples is equal to zero, while the alternative hypothesis is that the difference of location between the samples is different to zero. The analysis has been conducted at a significance level of 95%. In this study, this analysis has been conducted on both δD_C29_ and δD_C31_ values for two time-slices; 4600 to 2500 cal yr BP and 2500 cal yr BP to present. Mann-Kendall trend analysis, a nonparametric test, has been employed to identify the trends in time-series of δD_*n*-alkane_ and δ^13^C_*n*-alkane_ values of the Chachi core sediments. The null hypothesis of this non-parametric test is that there is no trend in the time-series. The alternative hypothesis is that there is a trend in the time-series. The analysis has been conducted at a significance level of 95%. The correlation between dependent and independent variables has been calculated using linear regression analysis. All the statistical analyses have been conducted using PAST (Paleontological Statistics) and Sigma Plot software (version 12.5).

## Results

### Molecular level characterization of the OM

**Fig 2:**
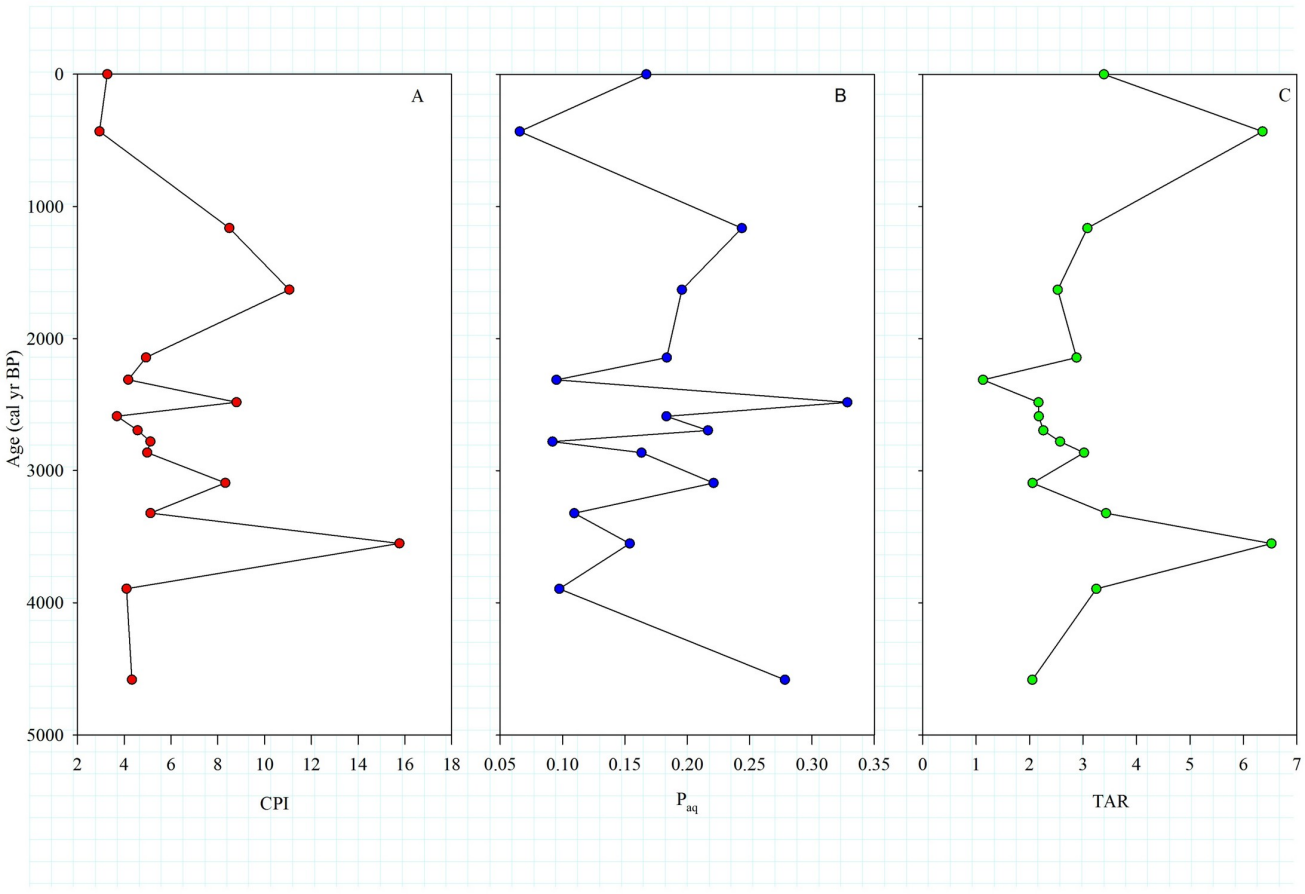
Down core profile of *n*-alkane indices in Chachi core sediments. (A) CPI, (B) P_aq_, and (C) TAR values for the Chachi core sediments.

The *n*-alkane distributions in leaves of modern plants showed the dominance (ca. 80% of total concentration) of C_29_ and C_31_ homologues (S1 Fig). On the contrary, the *n*-alkanes distribution in the core sediments displayed a distinct bimodal distribution with carbon numbers ranging from C_16_–C_35_ (S2 Fig). In the Chachi core sediments, total *n*-alkane concentration ranged from 120 μg g^-1^ to 700 μg g^-1^ and reached the maximal value at the topmost section of the core. The short-chain and long-chain *n*-alkanes accounted for ~ 40% and ~25% of the total *n*-alkane concentration respectively. The CPI values for the Chachi and Luna core sediments varied from 2.9 to 15.7 (Fig 2A) and 3.2 to 4.2 respectively. The P_aq_ values for the Chachi and Luna core sediments varied from 0.07 to 0.33 (Fig 2B) and 0.10 to 0.25, respectively. The TAR values of the Chachi sediments ranged from 1.1 to 6.5 (Fig 2C), whereas the Luna core sediments varied from 4.1 to 5.1.

### The *δ*D and *δ*^13^C values of long-chain *n*-alkane in modern plants

The δD_*n*-alkane_ and δ^13^C_*n*-alkane_ values of the modern vegetation from Banni region are listed in Table 1. The modern C_3_ vegetation in Banni region showed average δ^13^C_C29_ value of −33.6 ± 1.8 ‰ and δ^13^C_C31_ values of −35.5 ± 2.4 ‰ (n = 8, 1σ). The average δ^13^C_C29_ and δ^13^C_C31_ values of modern C_4_ plants are −22.4 ± 1.1 ‰ and −23.6 ± 1.6 ‰ (n = 5, 1σ) respectively. In C_3_ plants, δD_C29_ and δD_C31_ values varied from −173 ‰ to −98 ‰ (avg = −133 ± 25.1 ‰) and −198 ‰ to −132 ‰ (avg = −162 ± 24.7 ‰). The δD_C29_ and δD_C31_ values of C_4_ plants ranged between −218 ‰ to −144 ‰ (avg = −176 ± 28.9 ‰) and −219 ‰ to −150 ‰ (avg = −185 ± 28.6 ‰), respectively.

**Table 1:** Compound specific δD and δ^13^C values for the long chain *n*-alkanes of modern vegetation from Banni region. SD = standard deviation (1σ).

	Sample	δ^13^C_C29_ (‰, VPDB)	S.D.	δD_C29_ (‰, VSMOW)	S.D.	δ^13^C_C31_ (‰, VPDB)	S.D.	δD_C31_ (‰, VSMOW)	S.D.
**C**_**3**_ **plants**	*Solanum viarum*	-34.9	0.4	-122	4	-37.4	0.3	-198	2
	*Mangifera indica*	-34.9	0.4	-173	2	-37.2	0.3	-184	3
	*Eupatorium odoratum*	-32.7	0.2	-133	3	-37.6	0.3	-141	2
	*Salvadora persica*	-30.1	0.8	-116	4	-30.7	0.5	-132	1
	*Acacia nilotica*	-36.1	0.3	-165	5	-36.1	0.4	-189	2
	*Azadirachta indica*	-33.4	0.1	-141	4	-33.6	0.2	-154	3
	*Physalis minima*	-34.0	0.3	-123	3	-36.9	0.4	-143	1
	*Citrus lemon*	-32.6	0.4	-98	4	-35.0	0.3	-160	1
	**Average**	**-33.6 ± 1.8**		**-133 ± 25.1**		**-35.5 ± 2.4**		**-162 ± 24.7**	
**C**_**4**_ **plants**	*Cenchrus ciliaris*	-21.0	0.5	-218	4	-22.2	0.3	-150	2
	*Chloris barbata*	-22.8	0.3	-144	3	-23.7	0.4	-177	1
	*Panicum miliaceum*	-22.1	0.3	-177	1	-24.4	0.4	-209	4
	*Suaeda fruticosa*	-21.9	0.4	-187	4	-21.9	0.5	-219	4
	*Desmostachya bipinnata*	-23.9	0.3	-155	2	-25.8	0.2	-169	2
	**Average**	**-22.4 ± 1.1**		**-176 ± 28.9**		**-23.6 ± 1.6**		**-185 ± 28.6**	

### The *δ*^13^C value of long-chain *n*-alkanes in core sediments

**Fig 3:**
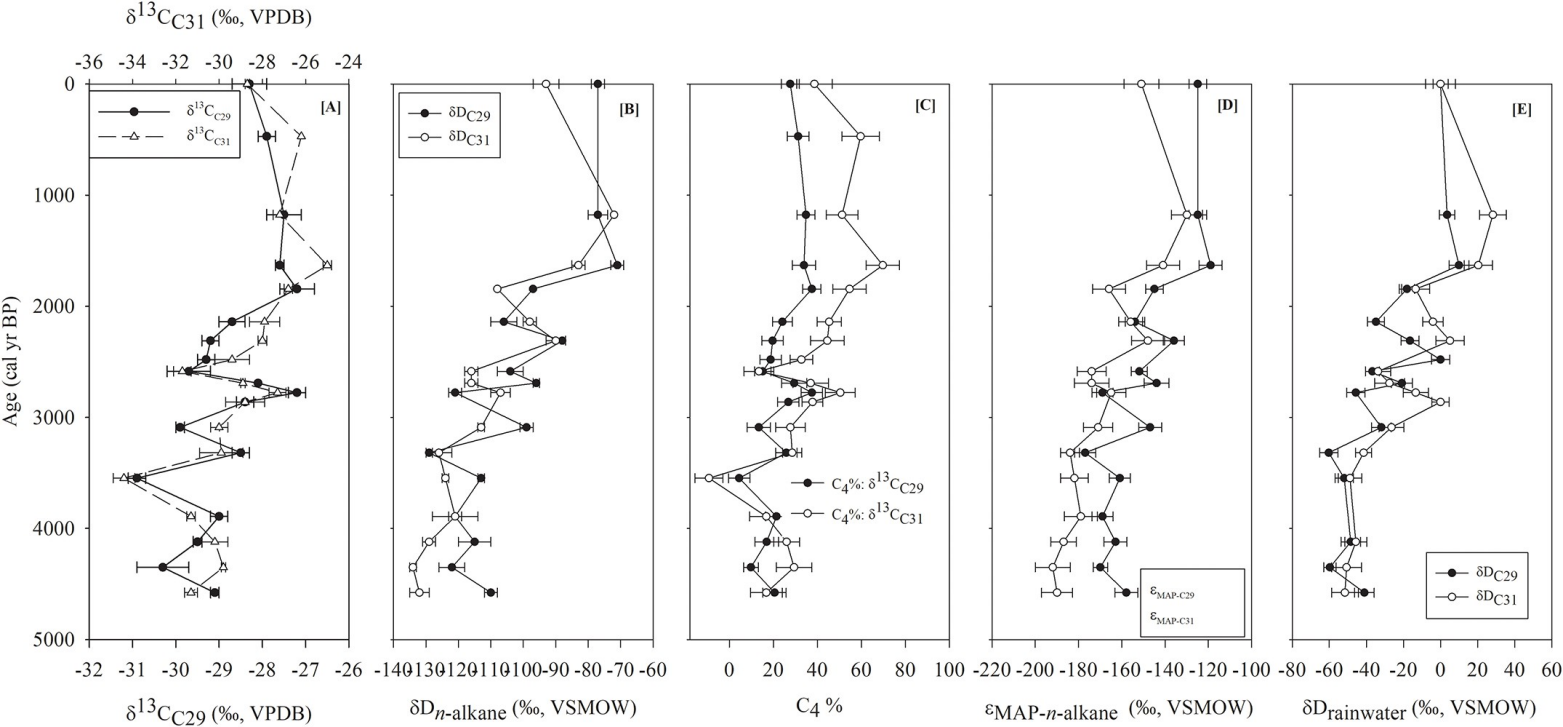
The isotopic profile and related calculations from the core sediments. [A] δ^13^C_*n*-alkane_ and [B] δD_*n*-alkane_ values for both *n*-C_29_ and *n*-C_31_ homologues. [C] Down-core calculated fractional C_4_ contributions from both *n*-C_29_ and *n*-C_31_ homologues. [D] Down-core apparent hydrogen isotopic fractionation (ε_app_) for each homologue calculated using the measured end-member compositions and calculated fractional C_4_ contributions. [E] Down-core reconstructed δD_rainwater_ values using the measured δD_*n*-alkane_ and calculated ε_app_ values for each homologue. The horizontal lines in the figure represent the error associated with the calculations.

The δ^13^C_C29_ and δ^13^C_C31_ values of the Chachi core sediments varied from −30.9 ‰ to −27.2 ‰ and −34.4 ‰ to − 25.0 ‰ respectively (Table 2). The δ^13^C_*n*-alkane_ values of both the homologues showed more negative values during 4600 to 2500 cal yr BP (Fig 3A). Afterwards, δ^13^C_C31_ values displayed a progressively increasing trend with maximum values at 1500 cal yr BP. Similar trends are also observed for the δ^13^C_C29_ values. The δ^13^C_C29_ and δ^13^C_C31_ values of the Chachi core sediments exhibit strong and significant positive correlation (R^2^ = 0.66, p < 0.05). The p-values of Mann-Kendall analyses for the both homologues are less than 0.05 and Kendall’s tau value for δ^13^C_C29_ and δ^13^C_C31_ values are 0.45 and 0.61. The δ^13^C_*n*-alkane_ values of Luna core sediments for the last ca. 1000 parallels the Chachi record (Fig 3). The δ^13^C_C29_ and δ^13^C_C31_ values of the Luna core sediments ranged from –25.3 to –27.7 ‰ and –23.9 to –28.0 ‰ respectively (Table 3).

**Table 2:** δD and δ^13^C values for the long chain *n*-alkanes of Chachi core sediments N.A. = not available/not determined. SD = standard deviation (1σ).

Sample	Depth	Age cal yr BP	δ^13^C_C29_ (‰, VPDB)	S.D.	δ^13^C_C31_ (‰, VPDB)	S.D.	δD_C29_ (‰, VSMOW)	S.D.	δD_C31_ (‰, VSMOW)	S.D.
Chachi sediments										
Ca-1	0	0	-28.3	0.4	-28.7	0.1	-77	2	-93	4
Ca-2	8	471	-27.9	0.2	-26.2	0.0	N.D.		N.D.	
Ca-4	20	1177	-27.5	0.4	-27.2	0.3	-77	3	-72	0
Ca-8	40	1631	-27.6	0.1	-25.0	0.2	-71	2	-83	2
Ca-12	60	1844	-27.2	0.4	-26.8	0.2	-97	0	-108	0
Ca-15	74	2140	-28.7	0.3	-27.9	0.7	-106	4	-98	2
Ca-17	82	2310	-29.2	0.2	-28.0	0.2	-88	0	-90	3
Ca-19	90	2480	-29.3	0.2	-29.4	0.8	N.D.		N.D.	
Ca-20	95	2586	-29.7	0.5	-31.7	0.4	-104	4	-116	2
Ca-21	100	2692	-28.1	0.0	-28.9	0.1	-96	1	-116	2
Ca-22	104	2777	-27.2	0.2	-27.3	0.4	-121	2	-107	3
Ca-23	108	2862	-28.4	0.2	-28.8	0.9	N.D.		N.D.	
Ca-24	112	3090	-29.9	0.1	-30.0	0.4	-99	2	-113	1
Ca-25	116	3319	-28.5	0.2	-29.9	1.0	-129	1	-126	4
Ca-26	120	3548	-30.9	0.2	-34.4	0.5	-113	1	-124	1
Ca-27	126	3892	-29.0	0.2	-31.3	0.2	-121	7	-121	2
Ca-28	130	4121	-29.5	0.1	-30.2	0.6	-115	5	-129	2
Ca-29	134	4350	-30.3	0.6	-29.8	0.1	-122	4	-134	1
Ca-30	138	4578	-29.1	0.1	-31.3	0.3	-110	2	-132	3

**Table 3:** δ^13^C values for the long chain *n*-alkanes of Luna core sediments. SD = standard deviation (1σ).

Lune sediments						
Sample	Depth	Age cal yr BP	δ^13^C_C29_ (‰, VPDB)	S.D.	δ^13^C_C31_ (‰, VPDB)	S.D.
Lb-1	3	10	-27.2	0.1	-27.5	0.1
Lb-2	9.5	109	-26.6	0.4	-27.3	0.5
Lb-3	15.5	274	-26.3	0.6	-25.8	0.8
Lb-4	21.5	438	-27.6	0.2	-28	0.4
Lb-5	27.5	603	N.D			N.D
Lb-6	33.5	670	-25.7	0.3	-25.9	0.1
Lb-7	39.5	737	-27.4	1.1	-27.7	0.3
Lb-8	45.5	804	-26.6	0.1	-26.2	0.7
Lb-9	51.5	871	-26.3	0.1	-25.9	0.3
Lb-10	57.5	937	-26.2	0.7	-26.6	0.6
Lb-11	62.5	951	-27.7	0.3	-27.5	0.1
Lb-12	67.5	962	-25.3	0.0	-23.9	0.5
Lb-13	72.5	973	N.D		N.D	
Lb-14	77.5	984	-25.8	0.6	-25.3	0.4
Lb-15	82.5	995	-27.3	0.2	-27.9	0.6

### The δD values of long-chain *n*- alkanes in core sediments

The p-values of Mann-Kendall analyses for the both homologues are less than 0.05 and Kendall’s tau value for δD_C29_ and δD_C31_ values are 0.72 and 0.76, respectively. The magnitudes of fluctuation in δD values were similar for both C_29_ and C_31_ homologues (Table 2). In most of the samples, the δD_C29_ values were more positive compared to δD_C31_ value. The most prominent feature of the curve is the shift towards positive values after 2500 cal yr BP (Fig 3B). Accordingly, we have compared the values for two time-slices; 4600–2500 cal yr BP (phase-1) and 2500 cal yr BP to present (phase-2). The average δD_C29_ and δD_C31_ values during the phase-1 were −113.0 ± 10.7 ‰ and −121.8 ± 8.8 ‰ (n = 10, 1σ) respectively. The average δD_C29_ and δD_C31_ values during phase-2 were −86.0 ± 13.5 ‰ and −90.7 ± 12.3 ‰ (n = 6, 1σ). The difference in the δD_C29_ (and δD_C31_) values for phase-1 and phase-2 has also been exhibited by Mann-Whitney test statistics. For both the homologues, we have found the p-values are less than significance level of 0.05 and thus reject the null hypothesis.

### The stable isotopic composition of modern precipitation in Banni region

Monthly-weighted averaged δD values are positively correlated (R^2^ = 0.9, p < 0.05) with the δ^18^O values of precipitation. The Local Meteoric Water Line for the Banni region is:

$$\delta \text{D}=7.6\times \delta^{18}\text{O}+7.9$$

(Fig 4A)

**Fig 4:**
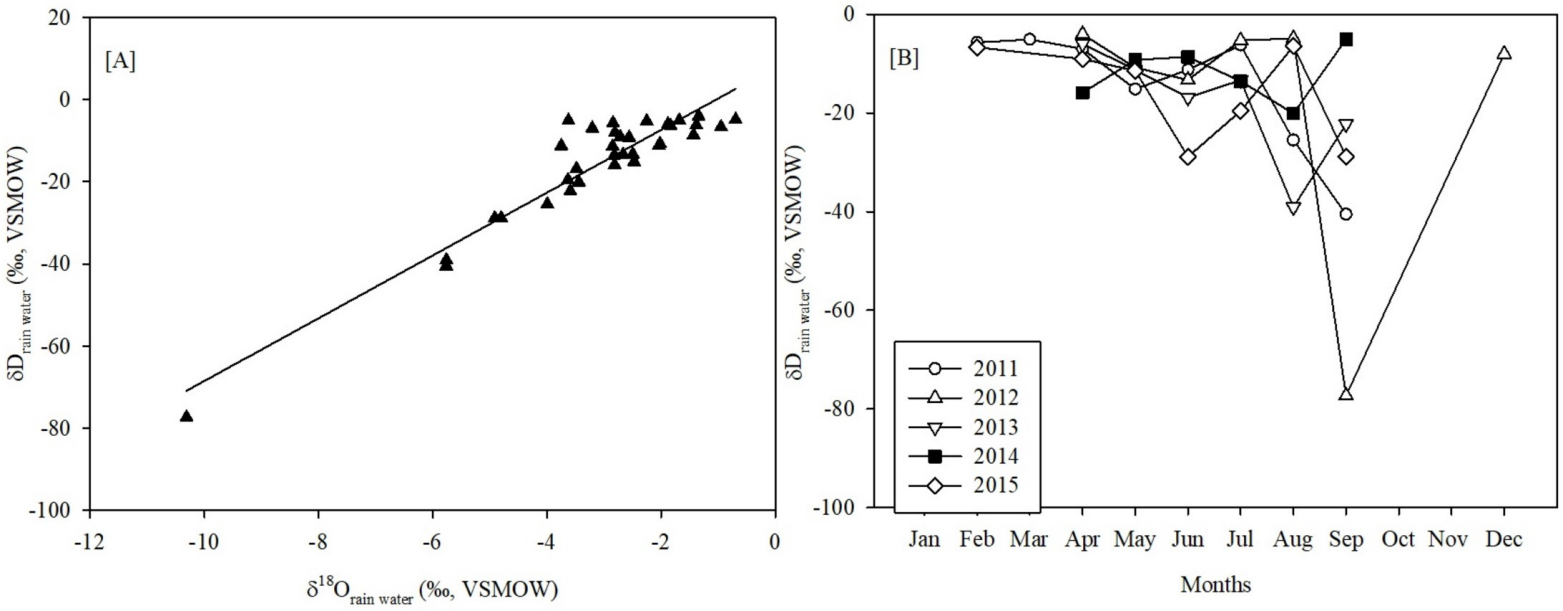
Isotopic variation of modern regional precipitation (IsoGSM2). (A) Local meteoric water line in the Banni region has been obtained using cross-plot between δD and δ^18^O values. (B) The monthly averaged δD values of precipitation become progressively depleted in heavier isotope with the establishment of the ISM.

Due to the significant correlation (R^2^ = 0.9), only the results of δD values of precipitation are further discussed. During 2011 to 2015, the δD values of precipitation varied from −4 ‰ to −77 ‰, and lower δD values coincide with the peak ISM precipitation (Fig 4B).

## Discussion

### Sources of organic matter

The *n*-alkane assemblage in the sediments is an effective biomarker tool for evaluating sources of organic matter due to the distinct carbon chain-lengths of terrestrial higher plants and microorganisms [26, 45]. In general, cuticular waxes of higher plants have a strong odd over even predominance in the long-chain *n*-alkanes and show CPI values >1, whereas the bacteria and algae are characterized by weak odd over even predominance and produce low CPI values (~1) [38]. Terrestrial higher plants are characterized by relatively higher abundances of odd-numbered long-chain *n*-alkanes, whereas microorganisms contribute short-chain *n*-alkanes [16, 36]. In Banni core sediments, the higher abundance of the short-chain *n*-alkanes (S2 Fig) indicates a significant contribution from microorganisms to the *n*-alkane pool in the sediments. On the other hand, peaks observed in the long-chain *n*-alkanes maximise at C_29_ and C_31_. The CPI values of both the sediment cores are consistent with the modern higher plants growing in the Indian subcontinent [27]. High CPI values (Fig 2A) suggest that long-chain *n*-alkanes in the sediments were derived from terrestrial higher plants. Further, the low P_aq_ values of Chachi core sediment indicate higher contribution of terrestrial biomass to the organic matter compared to aquatic productivity, and this observation is concordant with the TAR values (Fig 2B and 2C). Therefore, the long-chain δ^13^C_*n*-alkane_ values would be suitable for the paleovegetational reconstruction.

### Estimates of shifts from C_3_ to C_4_ plant type

A previous study from Banni grassland using bulk δ^13^C_org_ values showed that %C_4_ plant varied from 16% to 43% [12] (S1 Table). However, bulk δ^13^C_org_ values reflect contribution from multiple organic matter sources [16, 27], which are characterized by distinct isotopic composition. Furthermore, C_4_ plant derived components degrades faster compared to its C_3_ counterpart [46]. Therefore, paleovegetation reconstruction using the δ^13^C_org_ values may provide an erroneous estimate of %C_3_-C_4_ plants. The δ^13^C_*n*-alkane_ values can refine the interpretation of bulk δ^13^C_org_ data due to its resistance to biodegradation. Result of linear regression analysis shows that the δ^13^C_C29_ and δ^13^C_C31_ values of the Chachi core sediments exhibit a strong and significant positive correlation (R^2^ = 0.66, p < 0.05). The large spread in the δ^13^C_C29_ and δ^13^C_C31_ values (Fig 3A) shows substantial variations in the vegetation composition. The δ^13^C_*n*-alkane_ values are not only governed by the vegetation composition but also by physiological changes against moisture stress and δ^13^C value of atmospheric CO_2_ (δ^13^C_atmospheric CO2_) [42, 47, 48]. The results from the Indian subcontinent show that δ^13^C values of C_3_ plants change only −0.4 ‰ for every 100 mm increase in the precipitation amount [49]. Therefore, ca. 2350 mm change in the annual precipitation would be required to explain the observed spread in the δ^13^C_C31_ values (Table 2). Thus, we exclude moisture stress on the δ^13^C_*n*-alkane_ values as the estimated precipitation change in the region seems unrealistic. The δ^13^C_atmospheric CO2_ value has been declining rapidly in the recent decades as a consequence of the Suess effect (http://cdiac.ornl.gov/trends/co2/iso-sio/iso-sio.html). The modern δ^13^C_atmospheric CO2_ value (−8.2 ‰) is 1.7 ‰ lower compared to that in the Mid-Late Holocene period [47], and accordingly the end-member δ^13^C_*n*-alkane_ values of modern plants are corrected. The δ^13^C_*n*-alkane_ values are converted into %C_4_ plants based on mixing model, where end-member values are the average isotopic composition of the dominant C_3_-C_4_ plants in Banni region (Table 1).

In the core sediments, the δ^13^C_C31_ values correspond to 13% to 70% abundance of C_4_ plants, while δ^13^C_C29_ values indicate 5% to 38% abundance of C_4_ plants (Fig 3C). Chemotaxonomic investigations showed that C_4_ grasses in the savannah region synthesize more C_31_, whereas C_29_ is the dominant homologue in C_3_ dicots (S1 Fig) [50, 51]. The δ^13^C_C29_ and δ^13^C_C31_ values in the core sediments are believed to represent two distinct organic matter sources. Accordingly, in the present study, it has been assumed that δ^13^C_C29_ values of the sediment samples reflect the changes in the abundance of C_3_ trees, whereas δ^13^C_C31_ values indicate the changes in %C_4_ grasses. The measured δ^13^C_C31_ values provide higher estimation of C_4_ plants compared to both δ^13^C_org_ and δ^13^C_C29_ values (Fig 3C, S1 Table). Therefore, it can be suggested that the δ^13^C_C31_ values would be a more suitable proxy to infer vegetation change in the grassland setting.

The δ^13^C_C31_ value-based calculation indicates ca. 20% higher abundance of C_4_ plant during phase-2 compared to phase-1 (Table 4). Estimate of lower %C_4_ plant till 2500 cal yr BP is concurrent with the pollen and phytolith records which showed the dominance of woody taxa (i.e., C_3_ plants) during this interval [13]. The δ^13^C_*n*-alkane_ values from the Luna core sediments for the last 1000 cal yr BP also shows high %C_4_ plants in the Banni region. The trend in the δ^13^C_*n*-alkane_ values corresponds well with the vegetation record from central India [42].

**Table 4:** %C_4_ plants calculated using δ^13^C_C29_ and δ^13^C_C31_ values of the Chachi core sediments.

Sample Number	Age (cal yr BP)	C_4_% (δ^13^C_C29_)	Uncertainty	C_4_% (δ^13^C_C31_)	Uncertainty
Ca-1	0	27.7	4.1	38.7	8.0
Ca-2	471	31.3	4.9	59.7	8.5
Ca-4	1177	34.8	4.1	51.3	7.2
Ca-8	1631	33.9	5.3	69.7	7.6
Ca-12	1844	37.5	4.1	54.6	7.6
Ca-15	2140	24.1	4.5	45.4	5.5
Ca-17	2310	19.6	4.9	44.5	7.6
Ca-19	2480	18.8	4.9	32.8	5.1
Ca-20	2586	15.2	3.7	13.4	6.7
Ca-21	2692	29.5	5.7	37.0	8.0
Ca-22	2777	37.5	4.9	50.4	6.7
Ca-23	2862	26.8	4.9	37.8	4.7
Ca-24	3090	13.4	5.3	27.7	6.7
Ca-25	3319	25.9	4.9	28.6	4.3
Ca-26	3548	4.5	4.9	-9.2	6.3
Ca-27	3892	21.4	4.9	16.8	7.6
Ca-28	4121	17.0	5.3	26.1	5.9
Ca-29	4350	9.8	3.3	29.4	8.0
Ca-30	4578	20.5	5.3	16.8	7.2

### δD_C29_ value as a proxy for paleoprecipitation

The δD_*n*-alkanes_ values from plant leaf wax have been increasingly used for the reconstruction of paleohydrological conditions [52–54]. The δD variability in leaf *n*‐alkanes are mostly explained in terms of the changes in the δD values of regional meteoric water [54–59]. In addition, plant functional types and photosynthetic pathways can also affect the δD_*n*-alkane_ values [60–63]. In the Chachi core sediments, the δD_C29_ values differ by -14 ‰ to 22 ‰ to the δD_C31_ values of the same sample. Difference sources of *n*-alkane can be assumed to explain this offset [64] as net apparent hydrogen fractionation between source water (i.e., MAP) and lipid (ε_MAP-*n*-alkane_) of C_3_ dicots is ca. 30 ‰ more positive compared to that of C_4_ grasses [65].

Results from Banni grassland also show that ε_MAP-C29_ value for C_3_ plant is −113±25.1 ‰ while for C_4_ plant the value is −156±28.9 ‰ (Table 1). Similar differences have been inferred from C_31_ homologue as well. The difference in water absorption systems in plant forms plays an important role that cause more negative δD values in *n*-alkanes from grasses than those from woody plants. The grasses (C_4_) in the open ecosystem utilize water from deeper water sources (due to greater rooting depth of perennial grasses) compared to trees (C_3_) in woodland setting as woody plants draw most of their water from soil surface through radial and shallow roots [65]. The different water-usage strategies of C_3_ and C_4_ plants may lead to a significant offset in the ε_MAP-*n*-alkane_ (as well as δD_*n*-alkane_) values and required to be addressed when the vegetation changes are large (as in the case of the present study).

In this study, the proportion of C_4_ plants for each time-point has been calculated from the δ^13^C_C29_ and δ^13^C_C31_ values of the sediment samples (Table 3). Using the δ^13^C_*n*-alkane_ based estimates of vegetation changes, the weighted averaged apparent fractionation factor between δD_*n*-alkane_ and the δD_precipitation_ (ε_*n*-alkane_-_precip_) has been estimated using the following equation;

$$\varepsilon_{\text{n}-\text{alkane}-\text{precip}}=\text{A}\times \varepsilon_{\text{n}-\text{alkane}-\text{precip}}{}_{{(\text{C}}_{3}\text{plants})}+(100-\text{A})\times \varepsilon_{\text{n}-\text{alkane}-{\text{precip}(\text{C}}_{4}\text{plants}})$$

Where, A is the %C_3_ plants. The weighted averaged ε_*n*-alkane_-_precip_ values for each time-point have been presented in Fig 3D. Before the estimation of δD_precipitation_ values, it is required to calculate the hydrogen isotopic fractionation during organic matter transport from leaf-wax to soil. Using the above-mentioned equation, the δD_*n*-alkane_ value of modern leaf would be −125 ‰. On the other hand, the δD_C29_ value of the core-top sediment is −78 ‰, indicating incorporation of *n*-alkane into soil resulted in ca. 47 ‰ increase of the δD_C29_ value. Accordingly, the δD_*n*-alkane_ values for each time-point have been corrected [66]. The δD value of regional precipitation has been calculated using the following equation [67];

$${\delta \text{D}}_{\text{precipitation}}=\left[\frac{\left({\delta \text{D}}_{\text{n}-\text{alkane}(\text{corrected})}+1000\right)}{\left(\varepsilon_{\text{n}-\text{alkane}-\text{prec}}/1000)+1\right)}-1000\right]$$

**Fig 5:**
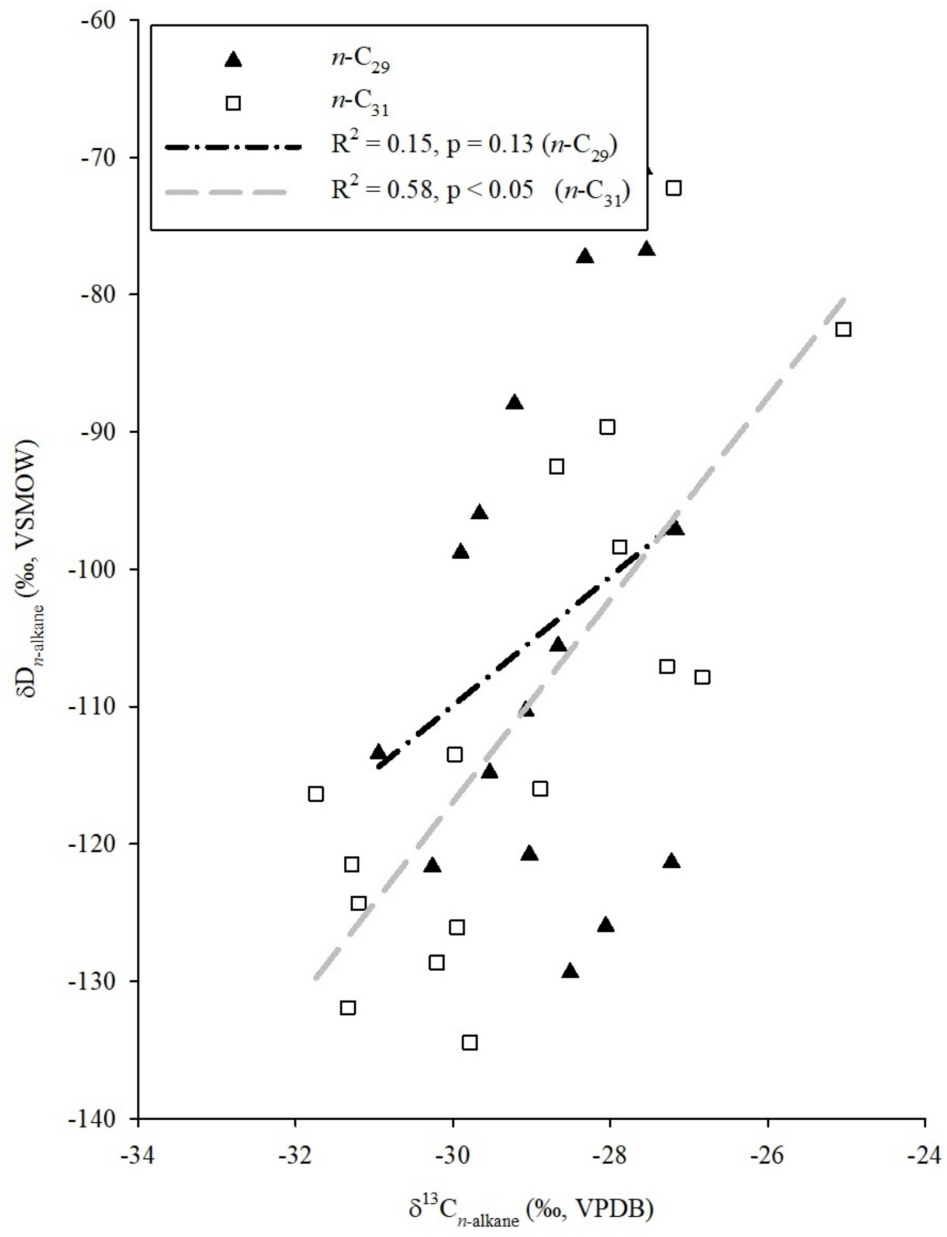
Regression analyses between δD_n-alkane_ and δ^13^C_n-alkane_ values for C_29_ and C_31_ homologues. Poor correlation (blue line) between the δD_*n*-alkane_ and δ^13^C_*n*-alkane_ values for C_29_ homologue indicates limited control of vegetation composition on the δD_*n*-alkane_ values. Strong and significant positive correlation (red line) between the δD and δ^13^C_*n*-alkane_ values for C_31_ homologue also suggests that change in vegetation type does not have influence on the δD_*n*-alkane_ values in collected samples.

The correlation analyses have been conducted between δ^13^C and δD values of both the homologues for further interpretation of paleohydrological conditions. Results of the linear regression analysis have shown strong and significant positive correlation (R^2^ = 0.58, p < 0.05, Fig 5) between δD_C31_ and δ^13^C_C31_ values. The vegetation change as primary driver of hydrogen isotopic variability of C_31_ homologue can result in negative correlation between δ^13^C_C31_ and δD_C31_ values. In contrast, the positive correlation indicates that the vegetation change has not influenced the hydrogen isotopic variations in C_31_ homologue. It has been further observed that the correlation between δD and δ^13^C values for C_29_ homologue is weaker and insignificant (R^2^ = 0.15, p = 0.13, Fig 5) suggesting that the δD values of this homologue are not influenced by the change from C_3_ plants to C_4_ grass [53]. Therefore, the δD_n-alkane_ values of both the homologues can be used as a surrogate to understand the paleohydrological conditions. In this study, the further discussion is based on the δD_C29_ values. The δD_precipitation(C29)_ values found to vary between 10 ‰ to −60.2 ‰ (Fig 3C).

In the low latitude tropical region, δD_precipitation_ values are negatively related to the precipitation amount (i.e., amount-effect) and this inverse correlation is often used to explain the variations in the δD_*n*-alkanes_ values [50, 51, 68, 69]. Instrumental record and back-trajectory computation of air-parcel show that precipitation in the Banni grassland is strongly seasonal (Fig 1D and 1E) with the dominant moisture contribution received from Arabian Sea. The seasonal difference in the net advection of moisture is reflected in the δD_precipitation_ values (Fig 4B). Seasonal precipitation amount peaks during ISM months and coincides with low δD_precipitation_ values, which indicates more negative δD_C29_ value of the sediment samples are representatives of high monsoonal precipitation events and vice-versa.

### Paleohydrological changes in Banni grassland

More negative δD_C29_ values till 2500 cal yr BP suggest an enhanced monsoonal precipitation in Banni. Little fluctuations (max. 10 ‰) show that the region had experienced minor changes in the precipitation amount during phase-1. Relatively stable and high precipitation during this phase accompanied by higher %C_3_ plants (Fig 3C). After 2500 cal yr BP, the δD_C29_ values gradually shift towards more positive values, and the aridification peaked around 1500 cal year BP. Regional aridity is also marked with the higher abundance of C_4_ grasses during this interval (Fig 3C). A negative excursion (ca. 10 ‰) after 1000 cal yr BP shows a slight increase in precipitation amount in the Banni region towards the present. Overall, it can be suggested that the Banni region had experienced a gradual shift from more mesic to more arid condition since the last 4600 cal yr BP. The δD_C29_ value based record is consistent with the interpretation made from δ^18^O_shell_ values in Banni core sediments [12].

### Critical role of fire, CO_2_ and precipitation on grassland stability

In general, low CO_2_ condition and frequent fire events promote higher %C_4_ grasses at the expense of C_3_ woody plant cover [70]. According to the CO_2_ crossover model [71], Banni with temperature > 30 °C, the threshold atmospheric CO_2_ level favoring C_4_ plants over C_3_ plants should be below 300 ppmv. The *p*CO_2_ reconstruction for the studied period showed that atmospheric CO_2_ level increased from 268 ppmv to 282 ppmv, a range favorable for the expansion of C_4_ plants. However, the trend of C_4_ plant expansion in Banni does not mimic the paleo-*p*CO_2_ curve and exhibits an anti-phase relationship in between 2500 to 1500 cal yr BP (S3 Fig). Therefore, *p*CO_2_ level alone cannot be attributed to the changing vegetation composition in Banni for the last 4600 cal yr BP.

The present study, in combination with published records, has attributed the increasing %C_4_ plants to the feedback mechanism between aridity, fire and vegetation composition. Increasing fire events (evidenced from Charcoal accumulation rate (CHAR)) from 2500 to 1500 cal yr BP helped to reduce C_3_ woody plant cover and favored the expansion of C_4_ grasses (S3 Fig). These fire events also promoted an arid understory microclimate and resulted in further drying in Banni. Continuous aridification helped fuel drying and led to more frequent fire events. In a nutshell, these effects have been enhanced by biogeophysical feedbacks; well-developed grasslands can favour the expansion of grasses by increasing light availability, reducing humidity and keeping high temperatures, all of which promote the growth of C_4_ plants and are capable of maintaining the grassland setting.

Expansion of C_4_ grasses can also impact the isotopic composition of the regional precipitation. Both IsoGSM2 model predictions and δD_n-alkane_ value reveal extremely high precipitation δD values from Banni region. The present study suggests the following mechanism to explain this distinct isotopic character: the transpiration of grasses is higher compared to forest trees [72, 73]. Although transpiration is a non-fractionation process, it indeed enriches the atmospheric vapor in heavier isotopes [73] and resultant precipitation has more positive isotopic composition.

### Regional comparison of climate data

**Fig 6:**
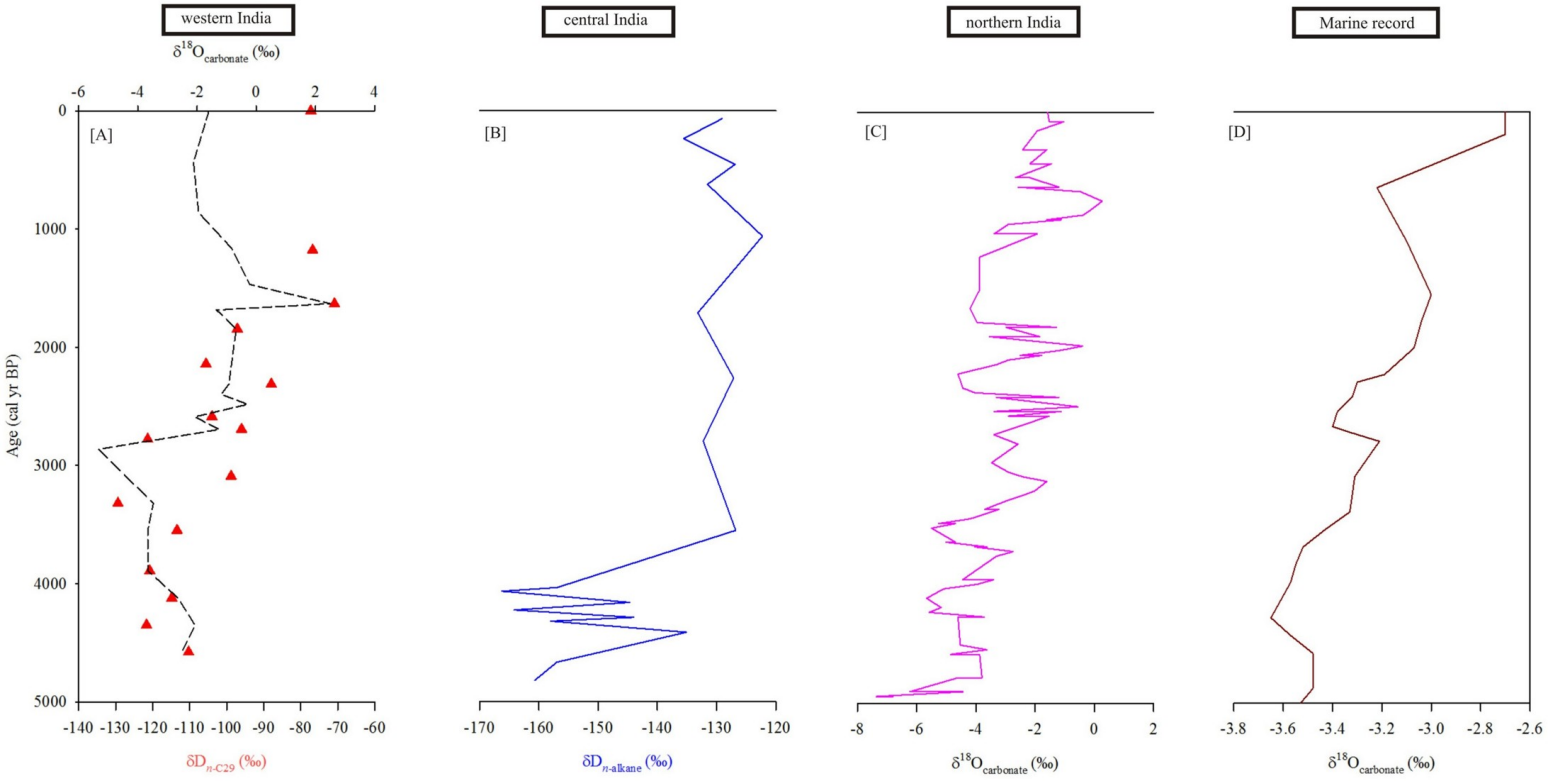
Compilation of the Mid-Late Holocene records from Indian monsoon realm. (A) δD_C29_ record from Banni region (this study) (B) δD record from Lonar Lake, central India [42], (C) δ^18^O_carbonate_ from Tso Moriri lake, NW Himalayas [11], and (D) δ^18^O_carbonate_ from northern Bay of Bengal [82].

The variations in precipitation in Banni grasslands for the last 4600 cal yr BP correspond well with published records from the ISM realm (Fig 6). The enhanced ISM precipitation in Banni during ca. 4600–2500 cal yr BP was synchronous with pollen and phytolith based investigations from Pariyaj lake in western India [74]. The geochemical proxies measured from the sediment core of an active mudflat of Diu Island in western India also indicate warm and humid conditions between 4105 and 2640 cal yr BP [75]. The results from Banni grassland are also corroborated by the geochemical and palynological analyses of relict mudflat from southern Saurashtra coast with wet climatic conditions and simultaneous occurrence of marginally high sea-level between 4710 and 2825 cal yr BP [76]. Likewise, the climatic reconstructions based on lake sediments from Nal Sarovar, central Gujarat [77] and Lunkaransar and Didwana Lakes of Rajasthan, western India [78, 79] observed a high lake stand during ca. 4680 and 3500 cal yr BP. The wetter conditions in Banni region during 4600–2500 cal yr BP is in good agreement with the record based on δ^13^C values of sedimentary leaf waxes from the core monsoon zone of India [9], and mineralogical and isotopic investigation on core sediments from Lonar lake, central India [10, 80]. This humid phase also matches with the geochemical investigations of core sediments retrieved from continental shelf in eastern India [81].

The onset of aridity in the Banni region after 2500 cal yr BP was in phase with lowering of the sea-level during 2825 and 1835 cal yr BP, as observed from the Saurashtra coast in western India [76]. The results are also in line with the high resolution record from NW India: Nal Sarovar lake [77]; Pariyaj Lake [74], Lunkaransar and Didwana lakes [78, 79]. The onset of aridity after 3 ka is also inferred in a tidal terrace sequence at the mouth of the Kharod River in western India [83]. A stalagmite record from peninsular India also indicates an abrupt climate change characterized by the decline of ISM around 2800 yr BP [84]. The findings of drier conditions in Banni region during ca. 2500–1000 cal yr BP is good accordance with the pollen record from NE India [85]; sedimentary leaf waxes from core monsoon zone [9] and geochemical data from eastern India [81]. More positive δD_*n*-alkane_ values of the Lonar lake sediments (central India) also provide evidences of the Late Holocene arid conditions (Fig 6B). The δ^18^O values of the carbonates from the Tso Moriri lake, northern India also demonstrates a decline in the intensity of ISM precipitation during the Late Holocene (Fig 6C). Our record from Banni grassland is also in line with the climatic reconstruction from northern Bay of Bengal [82] that confirms a gradual decrease in the ISM intensity during the Late Holocene (Fig 6D).

The record from the Banni grasslands matches with the evidence of precipitation changes and vegetation responses in the other tropical grasslands across the globe. For instance, terrestrial ecosystems in the Sahara responded to the weakening of African monsoon during the Mid-Late Holocene by a conspicuous reduction in tropical trees from nearly 4300 cal yr BP to the establishment of current day desert ecosystem by ca. 2700 cal yr BP [86]. Studies from the Manga Grasslands in northeastern Nigeria have also shown that climate was the major determinant of vegetation change during the Mid-Late Holocene with a shift towards more arid conditions until 1000 cal yr BP followed by wetter conditions towards the present [87]. Distinct biostratigraphical and sedimentological evidences of the shift towards drier climatic conditions during the Mid-Late Holocene were reported from multiple sites across Western Africa [88].

### Teleconnections governing long-term regional climate variability

**Fig 7:**
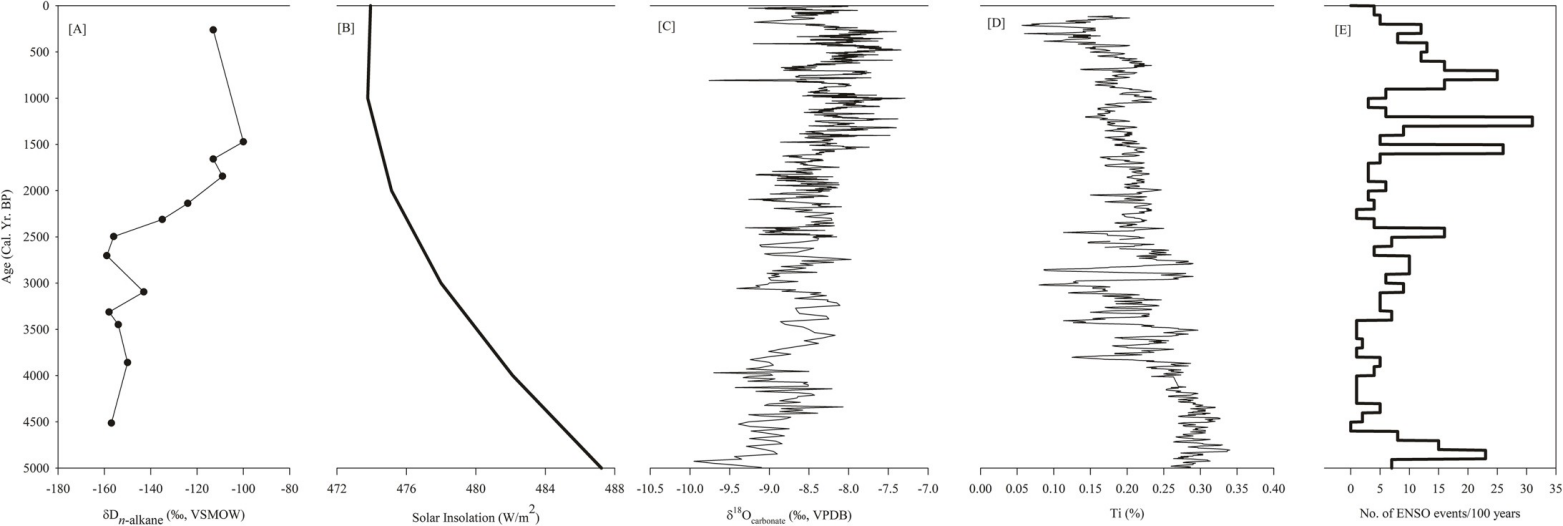
Compilation of Mid-Late Holocene records of terrestrial records across different monsoonal domains and forcing factors. (A) δD_C29_ record from Banni region (this study), (B) δ^18^O_carbonate_ from Heshang cave, EASM [96], (C) δ^18^O_carbonate_ from Liang Luar, AISM [90], (D) solar insolation curve from the northern hemisphere [91] and (E) frequency of ENSO activities [94].

As various components of the different monsoon systems are interconnected through feedback mechanisms, perturbation of any of those components can affect the global monsoonal circulation [89]. For instance, less boreal summer monsoonal precipitation is linked with the increased Australian-Indonesian summer monsoonal (AISM) precipitation [90]. Therefore, understanding of land-ocean-atmosphere interaction on a global scale requires quantitative data from different monsoon domains. Towards this, climate records from different monsoonal domains (Fig 7) have been compared in this study to understand the role of forcing factors that persist across Hemispheres. The mean δD_C29_ values of the Chachi core sediments are significantly different for the phase-1 and 2, and this difference is correlated with the mean position of the ITCZ which is driven by the solar insolation [91] (Fig 7D). Monsoonal precipitation in the Northern Hemisphere increases with northward migration of ITCZ, accompanying decreased precipitation in the Southern Hemisphere; and vice versa [92]. Paleoclimatic records showed that the Southern Hemisphere insolation became more seasonal compared to Northern Hemisphere in the Late Holocene. The mean position of ITCZ during the phase-2 is thus postulated as the driving mechanism for the drying trend in ISM and East Asian summer monsoon dominated region and wet condition in the Southern Hemisphere (Fig 7A and 7D).

Increasing ENSO activity couldn also be argued for the increase in the δD_C29_ values after 2500 cal yr BP. An investigation based on the modern instrumental record suggested that majority of droughts in the ISM dominated regions are linked with the warm phases of ENSO events (i.e., El-Nino) [93]. The modern ENSO regime was established 3000 to 4500 cal yr BP and El-Nino events significantly increased after 2500 cal yr BP [94] concurrent with the marked dry condition in the Banni region (Fig 7E). This pattern in the precipitation correlates well with the grain size data from EL Junco Crater Lake in the Galapagos Islands, inferred to reflect the higher frequency of El-Nino events [95]. Together position of ITCZ and ENSO activity appear to be linked to the inferred aridification in Banni region during the Late Holocene.

## Conclusion

Climate-driven changes in grassland ecosystems of the Indian Summer Monsoon realm have been investigated, for the first time using the isotopic composition of biomarkers in lake sediments. After ca. 2500 cal yr BP, a gradual increase in the δ^13^C_*n*-alkane_ values indicates the dominance of C_4_ grasses at the expense of C_3_ plants. Difference between the δ^13^C_*n*-alkane_ values indicates that each *n*-alkane homologue had different C_3_-C_4_ proportional contributions. In the lake sediments, C_29_ and C_31_ homologues were mainly derived from C_3_ plants and C_4_ grasses respectively. The present study has used the δD_C29_ values as a proxy of the isotopic composition of paleoprecipitation. Meteorological and modeling observations show that δD values of modern precipitation in the Banni region are inversely related to the precipitation amount. This ‘amount effect’ based reconstruction suggests a marked decrease in the ISM precipitation after 2500 cal yr BP. The observed precipitation pattern in the climate record from Banni region corresponds well with published records. Coupled influence of southward migration of ITCZ and higher frequency of warm ENSO events led to the decrease in the ISM precipitation after 2500 cal yr BP and helped in establishing the grassland ecosystems.

## Supporting information

S1 Fig Representative distribution of *n*-alkanes from the modern vegetation (trees and grasses) in Banni region. Pr and Ph represent the concentration of pristane and phytane isoprenoids in the vegetation.
(JPG)

S2 Fig Representative distribution of long-chain *n*-alkanes derived from Chachi core sediments. Pr and Ph represent the concentration of pristane and phytane isoprenoids in the sediments.
(JPG)

S3 Fig The dotted line represents the variations in the %Char in the Banni grassland for the last 4600 cal yr BP. The values of %Char has been taken from Pillai et al., 2017. The dashed line represents the variability of *p*CO_2_ concentration. The source of *p*CO_2_ dataset is Lüthi et al., 2008.
(JPG)

S1 Table The %C_4_ plants for the last 4600 cal yr BP has been estimated using bulk δ^13^C_org_, δ^13^C_C29_ and δ^13^C_C31_ values from the Chachi core sediments. Numbers in bold represent outliers.
(DOCX)
