# Supplementary figures and images for: Response of grassland ecosystem to monsoonal precipitation variability during the Mid-Late Holocene: Inferences based on molecular isotopic records from Banni grassland, western India
Source: PLoS One. 2019 Apr 17;14(4):e0212743. doi: 10.1371/journal.pone.0212743 (PMC6469751; doi:10.1371/journal.pone.0212743)

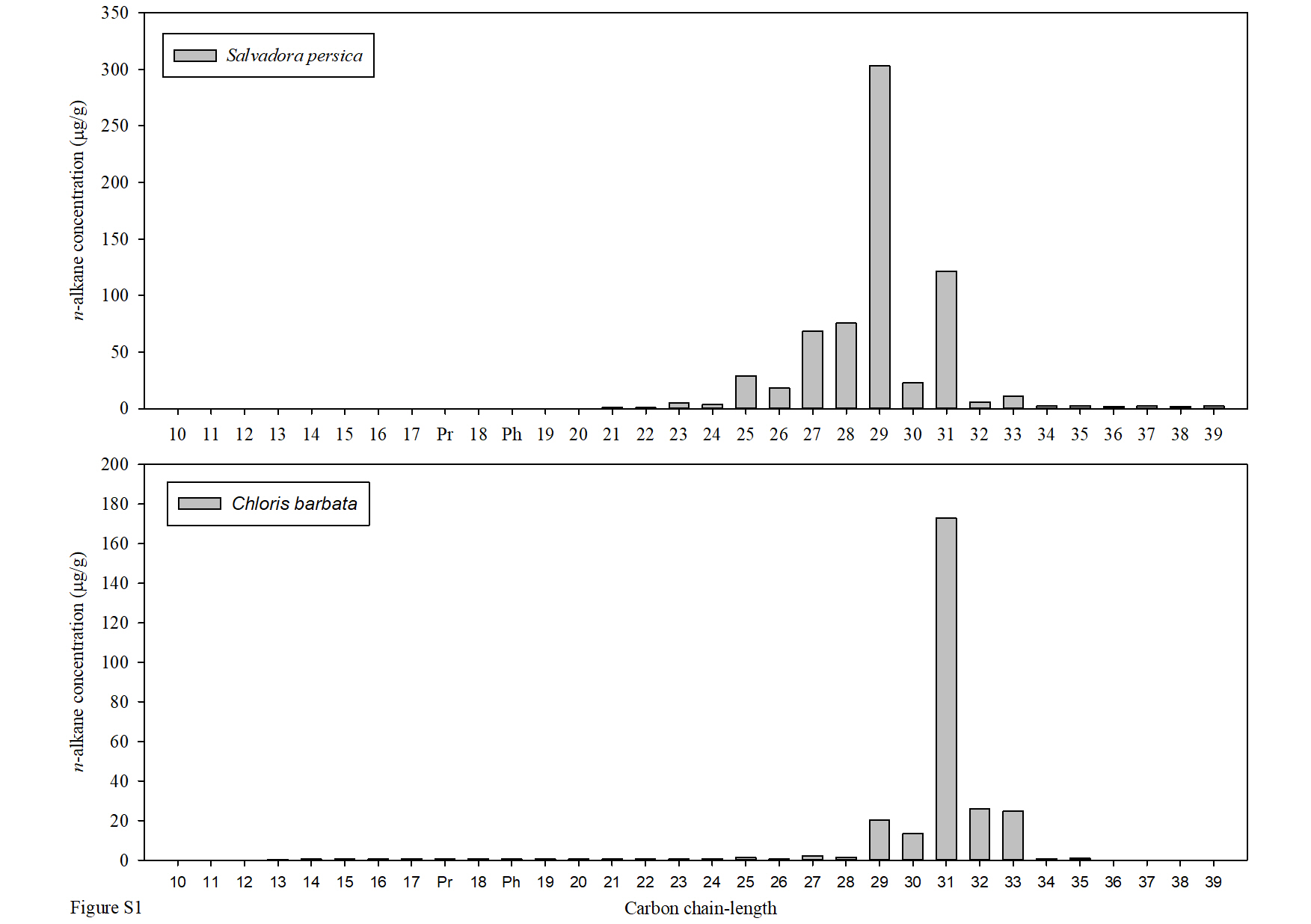

Supplement: S1 Fig — Pr and Ph represent the concentration of pristane and phytane isoprenoids in the vegetation. (JPG) [file pone.0212743.s001.JPG]

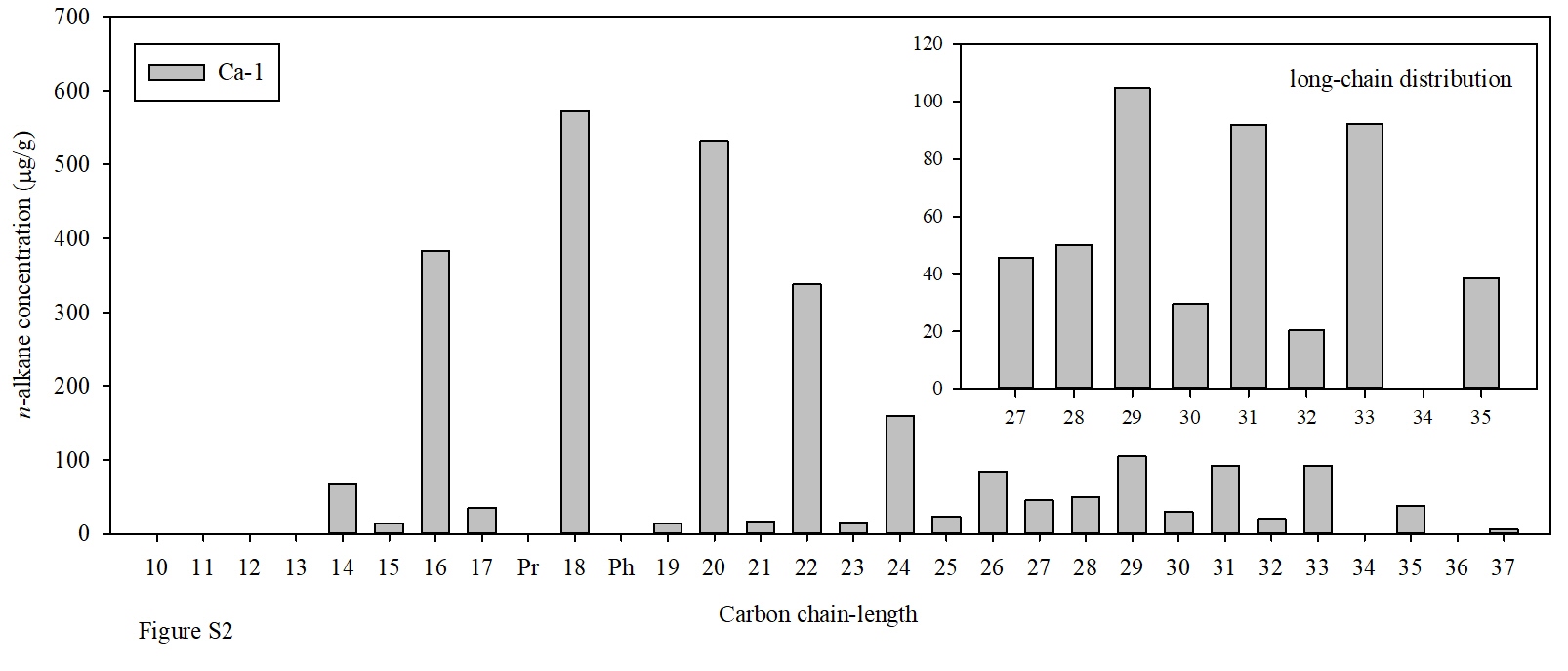

Supplement: S2 Fig — Pr and Ph represent the concentration of pristane and phytane isoprenoids in the sediments. (JPG) [file pone.0212743.s002.JPG]

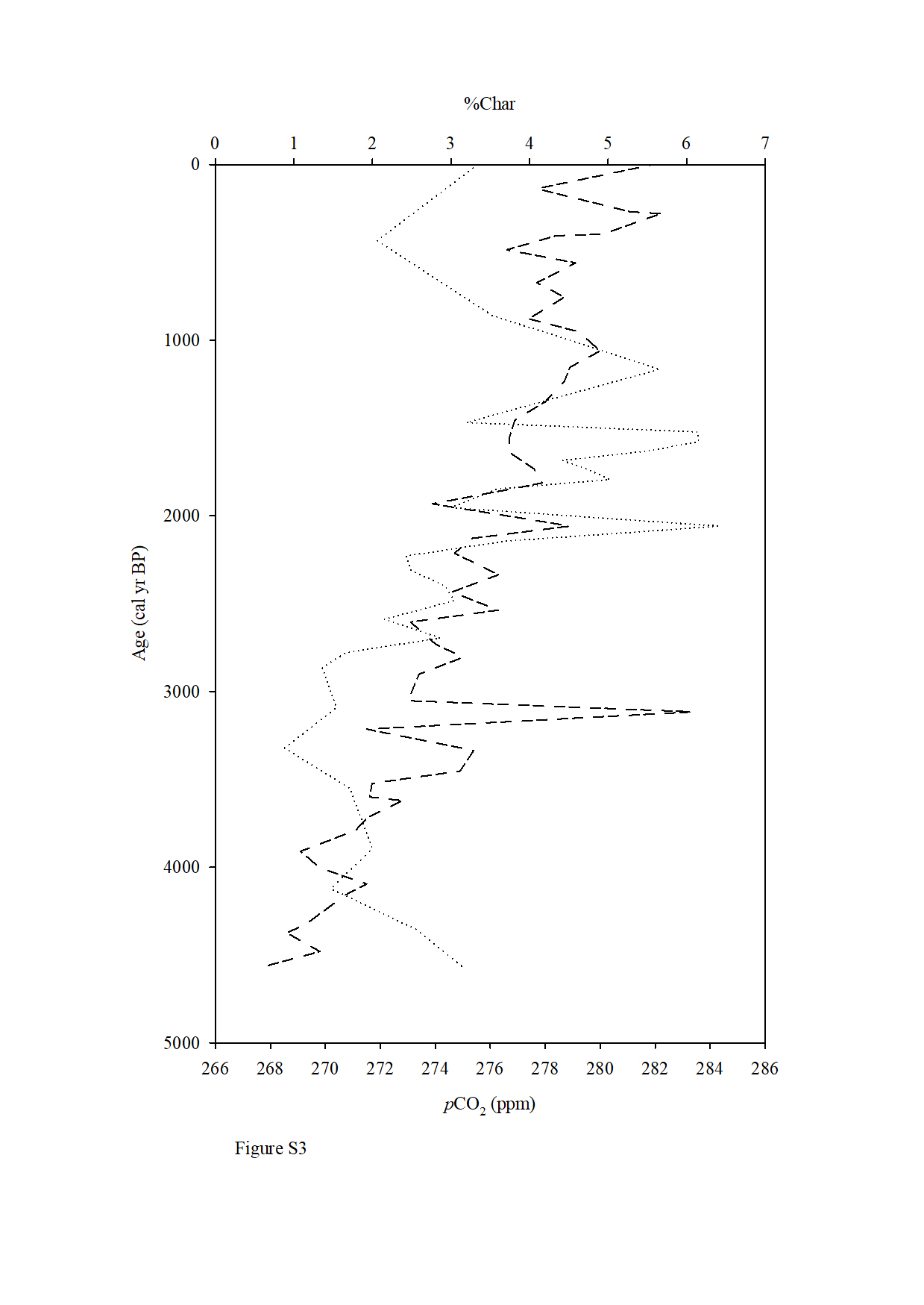

Supplement: S3 Fig — The values of %Char has been taken from Pillai et al., 2017. The dashed line represents the variability of pCO2 concentration. The source of pCO2 dataset is Lüthi et al., 2008. (JPG) [file pone.0212743.s003.jpg]
