# Supplementary material for: Response of grassland ecosystem to monsoonal precipitation variability during the Mid-Late Holocene: Inferences based on molecular isotopic records from Banni grassland, western India
Source: PLoS One. 2019 Apr 17;14(4):e0212743. doi: 10.1371/journal.pone.0212743 (PMC6469751; doi:10.1371/journal.pone.0212743)
Supplement: S1 Table — Numbers in bold represent outliers. (DOCX) [file pone.0212743.s004.docx]

Table S1:

| Chachi samples | Age (cal yr BP) | %C_4_ (estimated from bulk δ^13^C_org_) | %C_4_ (estimated from δ^13^C_C29_) | %C_4_ (estimated from δ^13^C_C31_) |
| --- | --- | --- | --- | --- |
| Ca1 | 0 | 31.0 | 27.7 | 38.7 |
| Ca2 | 432 | 23.8 | 31.3 | 59.7 |
| Ca4 | 1164 | 43.1 | 34.8 | 51.3 |
| Ca8 | 1630 | 35.2 | 33.9 | 69.7 |
| Ca12 | 1846 | 25.0 | 37.5 | 54.6 |
| Ca15 | 2143 | 19.0 | 24.1 | 45.4 |
| Ca17 | 2312 | 23.5 | 19.6 | 44.5 |
| Ca19 | 2482 | 26.2 | 18.8 | 32.8 |
| Ca20 | 2588 | **-29.**5 | 15.2 | 13.4 |
| Ca21 | 2694 | 16.6 | 29.5 | 37 |
| Ca22 | 2779 | 34.0 | 37.5 | 50.4 |
| Ca23 | 2864 | 29.8 | 26.8 | 37.8 |
| Ca24 | 3093 | 23.8 | 13.4 | 27.7 |
| Ca25 | 3322 | 19.3 | 25.9 | 28.6 |
| Ca26 | 3551 | **-14.8** | 4.5 | **-9.2** |
| Ca27 | 3894 | 33.7 | 21.4 | 16.8 |
| Ca28 | 4123 | 31.0 | 17 | 26.1 |
| Ca29 | 4353 | 28.0 | 9.8 | 29.4 |
| Ca30 | 4582 | 28.6 | 20.5 | 16.8 |
